# WeChat-Assisted Preoperative Health Education Reduces Burden of Care on Parents of Children with Simple Congenital Heart Disease: a Prospective Randomized Controlled Study

**DOI:** 10.21470/1678-9741-2020-0134

**Published:** 2021

**Authors:** Qi-Liang Zhang, Ning Xu, Shu-Ting Huang, Qiang Chen, Hua Cao

**Affiliations:** 1 Department of Cardiac Surgery, Fujian Maternity and Child Health Hospital, Affiliated Hospital of Fujian Medical University, Fuzhou, China.; 2 Department of Cardiovascular Surgery, Union Hospital, Fujian Medical University, Fuzhou, China.

**Keywords:** Health Education, Heart Defects, Congenital, Social Media, Parents

## Abstract

**Objective:**

The purpose of this study was to explore the clinical effect of preoperative health education based on the WeChat platform for parents of children with simple congenital heart disease.

**Methods:**

In this study, participants were randomly divided into an intervention group (WeChat group, n=40) and a control group (leaflet group, n=40) in our center. All parents were required to complete the Family Caregiver Task Inventory (FCTI) on the first visit and the Zarit Burden Interview (ZBI) and FCTI before the operation. Clinical, family and relevant data from children and parents were collected and subsequently analyzed.

**Results:**

Before the operation, the FCTI score and the ZBI score in the WeChat group were significantly lower than those in the control group (*P*=0.010, *P*=0.027, respectively). Compared to the FCTI score on the first visit, the preoperative status score was significantly lower in the WeChat group (*P*=0.008). The rate of loss to follow-up and complications in the WeChat group was also significantly lower than in the control group (*P*=0.003).

**Conclusion:**

Preoperative health education assisted by the WeChat platform for parents of children with simple congenital heart disease can effectively improve the parents' care ability and reduce the burden of care, preoperative complications and the rate of loss to follow-up.

**Table:** 

Abbreviations, acronyms & symbols	
**ASD**	**= Atrial septal defect**
**FCTI**	**= Family Caregiver Task Inventory**
**ZBI**	**= Zarit Burden Interview**

## INTRODUCTION

Simple congenital heart disease is one of the common congenital structural malformations, and surgical repair and transcatheter device closure are the main treatments. Atrial septal defect (ASD) is responsible for approximately 10-20% of congenital heart diseases, and the long-term results after treatment are satisfactory^[1,2]^. However, the clinical symptoms of some patients with simple congenital heart disease are not obvious, and they may choose intervention at a certain time rather than when the disease is first diagnosed. Many parents feel stressed or anxious for their children's preoperative home care and have difficulty in handling emergencies. Many studies have shown that the parents of children with congenital heart diseases suffer from more stress, anxiety and depression than parents of healthy children or children with other diseases, mainly because of the life-threatening and unpredictable disease and the lack of knowledge about the disease and care^[3,4]^. Therefore, we choose patients with ASD as our research object and provide adequate health knowledge to support these parents before operation so that children with ASD can receive better preoperative care.

In recent years, as the most popular mobile-based social media application in China, with 1.12 billion registered users, WeChat is now deeply integrated into the routine life of Chinese people. Like Twitter, Facebook, Skype and WhatsApp, WeChat supports numerous services for activities of daily living, including instant messaging (text, image and voice), sending red envelops, mobile payments, and many more. WeChat is gradually changing the channels through which people receive information. WeChat has also become a useful health education tool for managing diseases, such as cancer, chronic diseases and infectious diseases^[5-7]^. A study have shown that health education based on WeChat is more effective than traditional health education methods^[8]^. To our knowledge, there are no reports on the effect of preoperative implementation of WeChat-based health education for parents of children with simple congenital heart disease. This paper conducted a prospective randomized controlled study to evaluate the effectiveness of preoperative health education based on WeChat for parents of children with ASD.

## METHODS

The present study was approved by the ethics committee of Fujian Medical University, China, and adhered to the tenets of the Declaration of Helsinki. In addition, all patients signed the consent form before participating in the study.

### Calculation of the Study Sample Size

Based on the results of the pre-experiment and assuming that the alpha value was set at 0.05 with a power of 0.90, the required number of participants was calculated to be 36 in each group. Assuming a 10% missing rate, the total sample size was set as 80 (40 per group).

### Research Design

A prospective randomized controlled study was performed at our cardiac center in Fujian province, southeastern China. Clinical and family data of 80 children with ASD were collected from June 2017 to June 2018. All parents were required to complete the Family Caregiver Task Inventory (FCTI) on the first visit and the Zarit Burden Interview (ZBI) and FCTI before the operation. All clinical and family data are shown in Table 1. Inclusion criteria: 1) children diagnosed with ASD after birth; 2) selective treatment was set; 3) parents as primary caregivers; and 4) parents with smartphones who can use WeChat correctly. Exclusion criteria: 1) moderate and severe pulmonary hypertension; 2) need for emergency or scheduled surgery for children under 1 year; 3) other congenital heart diseases; 4) combined with other serious diseases; 5) refusal to participate in the study or follow-up plan.

**Table 1:** Demographic characteristics of patients and their parents in two groups.

	WeChat group	Control group	*P*
Age of patients(days)	22.6±16.5	26.1±17.6	0.646
Size of ASD (mm)	4.8±1.2	5.1±1.4	0.491
Pulmonary pressure (mmHg)	20.0±3.8	19.6±3.1	0.319
Intervention period (years)	2.6±1.2	3.1±1.0	0.688
Age of parents (years)			
<25 or 25	5	4	
26-30	17	14	
31-35	8	10	0.850
36-40	6	9	
>40 or 40	4	3	
Parents' education level			
Under high school	6	5	
High school	13	11	0.828
Junior college	12	16	
Bachelor degree or higher	9	8	
Living condition			
Rural area	26	28	0.633
City	14	12	
FCTI score on the first visit	32.8±5.6	32.2±4.9	0.312

### Data Acquisition

The researchers randomly divided eligible parents into the intervention group (WeChat group) and the control group (leaflet group) based on computer-generated random numbers. The researcher screened eligible parents for study and collected relevant data. They were also told not to disclose their group or share material with other parents.

### Intervention Methods

**Fig. 1:**
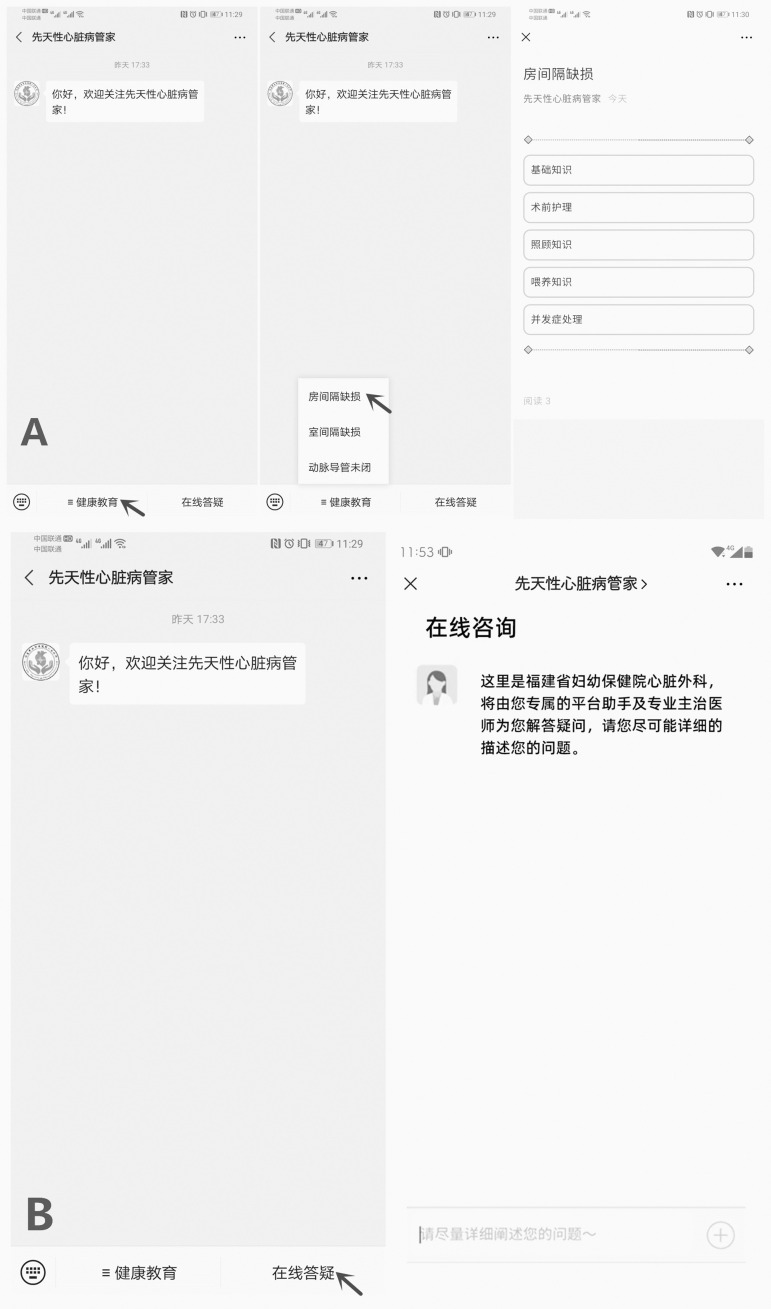
WeChat screen image including the platform to illustrate. (Chinese version). A) Education module. B) Question and answer module.

In the WeChat group, parents were provided health education and care guidance via the WeChat platform on their children's first visit, guided to join the WeChat platform, and taught how to use WeChat functions correctly and skillfully. The health education content in the WeChat group mainly included two parts: education module and question and answer module. The education module included the related knowledge of ASD disease, preoperative care, family care and feeding, and the management of complications. Parents could view it and learn at any convenient time. In the question and answers module, one medical staff of our team was on duty every day, and was online in the WeChat group from 6 pm to 10 pm to explain parents' problems, remember and supervise regular outpatient reviews and remind parents of the operation time. The medical staff also guided family members in the WeChat group to communicate, discuss, and share the care experience and actively encourage each other (Figure 1).

The parents in the control group obtained a leaflet on the first visit. The leaflet contained the same educational information as the intervention group and the time of reexamination and operation. They were also told to visit the hospital immediately in the event of an emergency.

### Research Tool

FCTI: the Chinese version of the FCTI scale that we used in this study was revised by Lee et al.^[9]^ based on the original FCTI scale by Clark et al.^[10]^ The scale consists of 25 items, including 5 dimensions: adaptation to roles of care, responding and providing assistance, addressing personal emotional needs, assessing family and community resources, and adjusting personal life and care needs. Each entry adopts the 3-point Likert scoring method: 0 points means not difficult, 1 point means difficult, and 2 points means extremely difficult. The total score of the scale is 50 points. The higher the score, the more difficulty the caregiver faces, and the less the ability of care.

ZBI: the ZBI scale was developed by Zait, and was translated into a Chinese version in 2006 by Wang et al.^[11,12]^It has 22 items, including two dimensions of individual burden and role burden. Item 22 is the overall assessment of the caregiving burden of caregivers. Each item is graded according to a 5-point Likert scale: "no", "occasionally", "sometimes", "often" and "always" were recorded as 0, 1, 2, 3, and 4 points, respectively. The higher the score, the heavier the care burden, with a total score of 88. A total score of <19 points indicates a light burden, 20 to 39 points indicate a moderate burden, 40 to 59 points indicate a heavy burden, and >60 points indicate a severe burden.

### Statistical Analysis

Continuous data were presented as the mean ± standard deviation and range. The normal distribution test was performed on all continuous data, and they followed the normal distribution. Clinical parameters between the two groups were compared with the independent samples t-test. The χ2 or Fisher's test was used to categorize the variables. A *P*-value of <0.05 was considered statistically significant.

## RESULTS

There was no significant difference between the two groups in baseline data. The results of the FCTI scale showed no significant difference between the two groups at the first visit (32.8±5.6 *vs*. 32.2±4.9, *P*=0.312). These data indicated that the two groups were homogeneous and comparable.

All patients in the WeChat group completed the follow-up process, while 8 patients (20.0%) in the control group were lost to follow-up, and the difference was significant (*P*=0.003). The FCTI score in the WeChat group was significantly lower than in the control group before the operation (15.8±4.5 *vs*. 27.3±7.5, *P*=0.010). Compared to the FCTI scores on the first visit, the WeChat group scores were significantly lower before the operation (*P*=0.008). The control group scores were also reduced, but the difference was not significant. The ZBI score in the WeChat group was also significantly lower than in the control group before the operation (23.8±8.3 *vs*. 43.8±13.7, *P*=0.027). The complications mainly included pulmonary infection and growth and developmental delay. The incidence of pulmonary infection and delayed growth and development in the WeChat group was significantly lower than in the control group (*P*=0.040, *P*=0.043) (Table 2).

**Table 2:** Comparison of preoperative care ability scores, burden of care scores, loss to follow-up and complications between the two groups.

	WeChat group	Leaflet group	*P*
FCTI score before the operation	15.8±4.5*	27.3±7.5	0.010
ZBI score before the operation	23.8±8.3	43.8±13.7	0.027
Loss to follow-up	0	8(20.0% 8/40)	0.003
Complications			
Pulmonary infection	3(7.5% 3/40)	8(25.0% 8/32)	0.040
Growth and development lag	2(5.0% 2/40)	6(18.8% 6/32)	0.043

* Shows that, compared to the first visit, *P*<0.05.

## DISCUSSION

ASD is one of the most common congenital heart diseases, and the majority of children are treated with elective surgery and intervention after 2-3 years of age^[1]^. Because of the left-to-right shunt of the ASD, the children's growth, development and physique may be worse than in normal children, and there is still a long time from the discovery of the disease to the operation; hence, many parents feel pressure and difficulty in the family care of the children before the operation^[13]^. Children's illness and hospitalization are the source of family crisis and parental anxiety^[14,15]^. Parental anxiety is mainly due to the lack of knowledge and information about the disease and care^[16-18]^. Although health education and various educational materials are provided to patients' parents at the outpatient service and inpatient wards, the results are not satisfactory, and most parents still have poor knowledge about the children's family care. In addition, due to the uneven distribution of medical resources, advanced medical treatment is mainly concentrated in large cities, and the basic medical level in rural areas is backward, especially in China. Professional knowledge of cardiac surgery is even more inadequate in rural areas, but the parents live mainly in the countryside. In this study, 67.5% of the children lived in rural areas, and their parents needed to go to hospitals in the big city to solve their children's health problems. They often line up all night, but only receive counseling services for a few minutes, which greatly increases the burden of care, the financial burden and the cost of time. This usually leads to patient dissatisfaction. Therefore, it is essential to develop new health education strategies for the children's parents, to provide them with continuous medical support and improve their knowledge of childcare.

In recent years, many different social media platforms have been widely used in health management and education of some chronic diseases, such as diabetes, hypertension, depression and coronary heart disease, to improve the clinical effect and reduce the anxiety of patients and their families^[19-21]^. WeChat is the most popular social media software in China, with more than one billion registered users, and is now deeply integrated into the daily life of the Chinese people. Many studies have shown that, as a health education tool for disease management, WeChat is more effective in reducing time and economic costs, improving treatment compliance, reducing patient complications, increasing follow-up rates and improving patients' condition than traditional methods^[5-7,22-24]^. Feng et al.^[6]^reported that WeChat services can improve adherence to corticosteroid nasal spray treatment for chronic rhinosinusitis after functional endoscopic sinus surgery. Li et al. used the official WeChat account to perform health education intervention for the prevention and treatment of malaria among non-immune travellers and expatriate workers and proved to be an effective, sustainable, feasible, and well-accepted strategy for improving health education on malaria^[7]^.

In our study, the WeChat platform was applied to the health education of parents of children with ASD. At the first appointment, the doctor instructed the parents to join the WeChat group and taught them to use the WeChat function correctly and skillfully. Parents can learn some professional knowledge about preoperative nursing, care and feeding, and management of complications for ASD from the WeChat education module anytime and anywhere. We also regularly publish popular science articles on preoperative care for simple congenital heart disease for parents to read. When they have problems, they can consult and communicate with other experienced parents in the WeChat group, and they can also consult a professional medical staff from 6 pm to 10 pm every day. Parents can receive professional support more easily and continuously through WeChat. In this study, compared to the control group, the FCTI and ZBI scores in the WeChat group both showed clear advantages. Patient complications (pulmonary infection and delayed growth and development) in the WeChat group were significantly less frequent than in the control group. It can be inferred that the implementation of health education through the WeChat platform could effectively improve the care ability of parents of children with ASD and reduce their burden of care, which allowed children to receive better preoperative care and reduce preoperative complications.

All patients in the WeChat group completed the follow-up, while eight patients in the control group were lost to follow-up. The loss to follow-up rate in the WeChat group was significantly lower. Through the continuous, low-cost, direct and interactive WeChat platform, parents can be reminded to participate in follow-up, which can effectively prevent the family members from losing contact with the doctor. However, patients in the control group only received an educational leaflet with the date of follow-up after the first visit. From the first visit to the operation, which can be at least one year, parents were prone to ignore the disease and forget to go to the hospital in time because the children had no symptoms.

There are still some limitations in this paper. First, due to unstable internet support and difficult access to WeChat, especially in rural areas of China, some qualified parents were not recruited. Second, this is a single-center study; larger, multicenter, and longer-term studies may yield different results. There may be some deviation in the selection of patients and the collection of research data, but we believe that this study still has some clinical significance. Finally, only patients with ASD were included in the study. Although the treatment and prognosis of several simple congenital heart diseases were similar, the conclusion of the corresponding study might be the same, but we hope to complete an additional study involving other types of simple congenital heart diseases.

## CONCLUSION

WeChat-assisted preoperative health education for parents of children with simple congenital heart disease can effectively improve the parents' care ability and reduce the burden of care, preoperative complications and the loss to follow-up rate. The implementation of health education through WeChat is convenient, fast, inexpensive and can achieve interactive communication, in addition to being worthy of clinical recommendation.

**Table:** 

Authors' roles & responsibilities	
Q-LZ	Substantial contributions to the conception or design of the work; or the acquisition, analysis or interpretation of data for the work; drafting the work or revising it critically for important intellectual content; final approval of the version to be published
NX	Substantial contributions to the conception or design of the work; or the acquisition, analysis or interpretation of data for the work; final approval of the version to be published
S-TH	Substantial contributions to the conception or design of the work; or the acquisition, analysis or interpretation of data for the work; final approval of the version to be published
QC	Substantial contributions to the conception or design of the work; or the acquisition, analysis or interpretation of data for the work; drafting the work or revising it critically for important intellectual content; final approval of the version to be published
HC	Substantial contributions to the conception or design of the work; or the acquisition, analysis or interpretation of data for the work; drafting the work or revising it critically for important intellectual content; final approval of the version to be published
